# Outcome measurements following palatal soft tissue graft harvesting: A review

**DOI:** 10.4317/jced.57625

**Published:** 2021-05-01

**Authors:** Violeta Malpartida-Carrillo, Pedro-Luis Tinedo-Lopez, Maria-Eugenia Guerrero, José-Luis Huamani-Echaccaya, Mutlu Özcan, Cassiano-Kuchenbecker Rösing

**Affiliations:** 1 DDS, MSc. Professor, School of Stomatology, Universidad Privada San Juan Bautista, Lima, Perú; 2 DDS, PhD. Professor, Department of Medico Surgical Stomatology, Faculty of Dentistry, Universidad Nacional Mayor de San Marcos, Lima, Perú; 3 DDS, MSc. Professor, School of Stomatology, Universidad Privada San Juan Bautista, Ica, Perú; 4 DDS, PhD. Professor, Center of Dental Medicine, Division of Dental Biomaterials, Clinic for Reconstructive Dentistry, University of Zurich, Zurich, Switzerland; 5 DDS, PhD. Professor, Department of Periodontology, School of Dentistry, Federal University of Rio Grande do Sul, Porto Alegre, Brazil

## Abstract

**Background:**

Free gingival graft (FGG) and connective tissue graft (CTG) are two of the most commonly techniques performed in periodontal and peri-implant plastic surgery. Although several outcome measurements have been proposed for evaluation of palatal wound healing and patient morbidity, a comprehensive review about these variables is lacking. The objective of this review is to present comprehensive information about outcome measurements related to postoperative palatal wound healing and postoperative patient-reported morbidity after FGG or CTG procedures.

**Material and Methods:**

An electronic search of English language dental literature in the Medline database via PubMed access was conducted from May 1994 to May 2020 following the PRISMA guidelines. Electronic search strategy complemented by hand search of impacting related dental journals, and the reference list of all included studies were used to complete data collection considering only clinical trials. Finally, inclusion criteria were applied to identify articles after full-text evaluation.

**Results:**

A total of 111 articles were identified. After the exclusion of 34 articles based on title and abstract evaluation, 77 articles were full text screened. Following, 46 articles were excluded since they evaluated other surgical areas. Finally, 31 studies were selected and included for final evaluation. Outcome measurements were classified in variables collected by indexes and systems assessed professionally and patient-centered measurements. Visually-assessed measurements include indexes, photographs, bleeding and use of laboratory-aided measurements. Patients-centered outcomes comprise pain, discomfort and quality-of life, among others.

**Conclusions:**

The most commonly used outcome measurements related to postoperative palatal wound healing are hydrogen peroxide test, tissue color match, visual inspection, and bleeding evaluation. Pain perception, analgesic consumption, discomfort sensation, burning sensation, and changes in feeling habits are the most commonly used outcome measurements related to postoperative patient-reported morbidity.

** Key words:**Morbidity, patient comfort, periodontics, tissue harvesting, wound healing.

## Introduction

Esthetic driven-dentistry has become an integral part in periodontal and peri-implant treatment. Several soft tissue grafting techniques such as free gingival graft (FGG), connective tissue graft (CTG), grafts combining these two modalities, and pedicle grafts have been used for re-establishing the keratinized tissue width (KTW), augmenting tissue thickness, correcting mucogingival deformities, and improving esthetics, at teeth and dental implant sites (1,2).

Despite the introduction of the above aforementioned techniques, the FGG and the CTG are two of the most commonly performed techniques in periodontal and peri-implant plastic surgery that use a safety zone of the hard palate as a common site for harvesting soft tissue grafts (3). These procedures have gained popularity due to the reasonably straightforward surgical procedure and exceptionally predicTable approach with reliable results (4). However, the main drawback of the FGG and the CTG procedures is patient morbidity at the palatal donor site that heals with primary or secondary intention approximately 2-4 weeks after the surgery (5), and requires a longer healing period with more patient discomfort and pain (6).

According to the literature, the current understanding about palatal wound healing is geared toward the study of periodontal dressing varieties to accelerate the healing process and to reduce prolonged pain and bleeding. Therefore, several materials such as hemostatic agents (absorbable gelatin sponge, absorbable collagen dressing, oxidized regenerated cellulose) (7-9), growth factors (10), and medical plant extracts (6), have been used to investigate their benefits on the palatal donor site. On the other hand, several methods for outcome evaluation such as modified Early-Wound Healing Index (11); visual inspection of color, contour, and texture changes (10); records of sensibility disorders, loss of sensor, changes in feeding habits, and pain perception (12) have been considered to evaluate healing characteristics and patient discomfort after FGG or CTG procedures. In addition, numerical and verbal rating scales have been included in postoperative questionnaires handed to patients (6,9-11). However, to the best of the authors’ knowledge, no studies have reported these methods in an organized manner and this would be an important tool for clinicians that perform periodontal and peri-implant plastic surgeries in order to adequately control the postoperative morbidity of the patients who undergo FGG or CTG procedures.

Hence, the aim of this review is to analyze and present comprehensive information about the outcome measurements evaluation related to postoperative palatal wound healing and postoperative patient-reported morbidity after undergoing FGG or CTG.

## Material and Methods

The Medline database via PubMed access was comprehensively searched from May 1994 to May 2020 according to the Preferred Reporting Items for Systematic Review and Meta-Analysis (PRISMA) guidelines (13). The search strategy was carried out using the following terms: “palatal”, “gingival graft” and “patient outcomes”. These terms were combined with the Boolean operators “AND” and “OR”. The final strategy was: ((palatal) AND ((free gingival graft) OR (connective gingival graft))) AND (patient outcomes). In addition, a hand search in dental journals (Journal of Periodontology, Journal of Clinical Periodontology, Clinical Oral Implants Research, International Journal of Oral and Maxillofacial Implants, Clinical Implant Dentistry and Related Research, International Journal of Periodontics and Restorative Dentistry) was also performed and the reference lists of all included articles were manually cross-referenced to complete data collection. Two reviewers (VMC and PLTL) screened the titles and abstracts for eligibility. The two reviewers were able to reach a consensus for all articles accepted for inclusion.

The inclusion criteria for selecting articles were clinical trials published in English that included outcome measurements evaluated after FGG or CTG harvesting from the palatal donor site related to the wound healing and the patient morbidity after the procedures. Animal studies, *in vitro* studies, observational studies, case reports, case series, reviews, and narrative studies were not considered.

## Results

**Figure 1:**
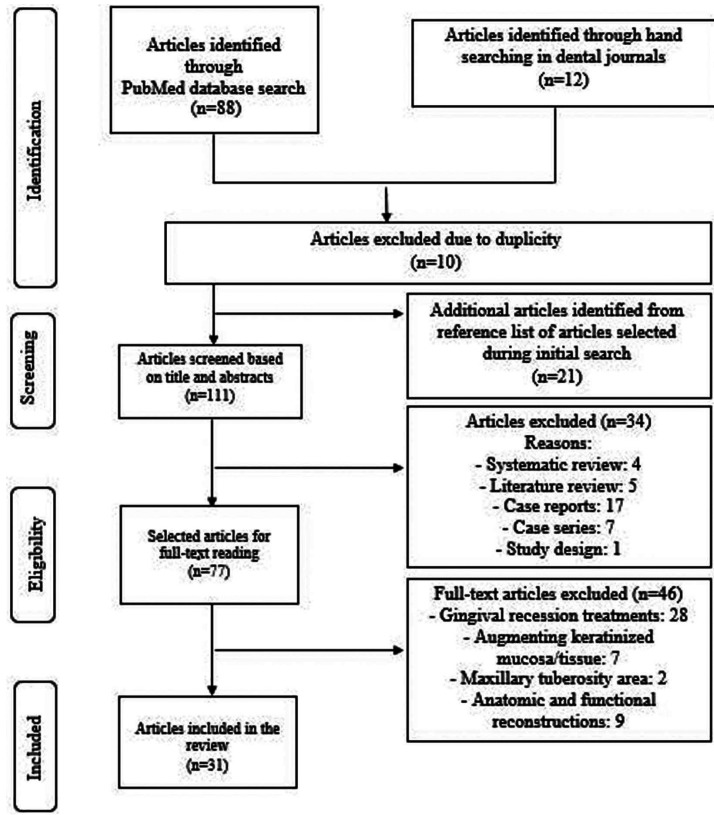
Flowchart of the review to identify included studies.

A total of 88 articles were identified after the initial search in PubMed database and 12 articles by hand searching. From these, 10 articles were discarded due to duplicity. Twenty-one articles were added from the reference list of articles selected during initial search and 111 articles were screened based on title and abstracts. However, 34 publications were excluded after title and abstracts evaluation, leaving 77 articles whose full text were screened. Then, 46 articles were excluded based on the inclusion criteria because different surgical areas for healing outcome were evaluated (recipient sites). Finally, 31 studies were selected and included for final evaluation. Figure 1 shows the flowchart of the review to retrieve the included studies.

Among the methods for outcome measurements evaluation related to postoperative palatal wound healing ( Table 1,  Table 1 cont.), the organized articles were distributed considering different objective measurements such as index and scales, wound epithelialization test, wound visual clinical healing, wound photographic healing, bleeding evaluation, cytological analysis, laboratory analysis, and histological examination. Fick *et al.* (11) Isler *et al.* (14) and Alpan *et al.* (15) described the use of the modified Early-Wound Healing Index (EHI) for postoperative healing evaluation after CTG considering five different degrees related to primary and secondary wound closure. This index comes from the original EHI proposed by Wachtel *et al.* (16) for periodontal intrabony defects postoperative healing evaluation. Another index is the modified Landry Wound Healing Index (WHI) reported by Isler *et al.* (14) Samani *et al.* (17) and Ustaoglu *et al.* (18) for postoperative healing evaluation after FGG considering parameters from very poor to excellent healing. Moreover, Samani *et al.* (17) reported the modified Manchester Scale for Clinical Scar, specifically performed after FGG procedures.

**Table 1:**
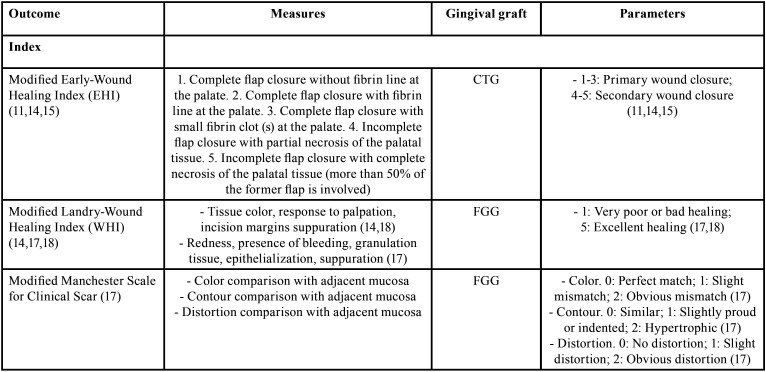
Description of outcome definitions and measures related to postoperative palatal wound healing retrieved in the literature.

**Table 1 cont.**
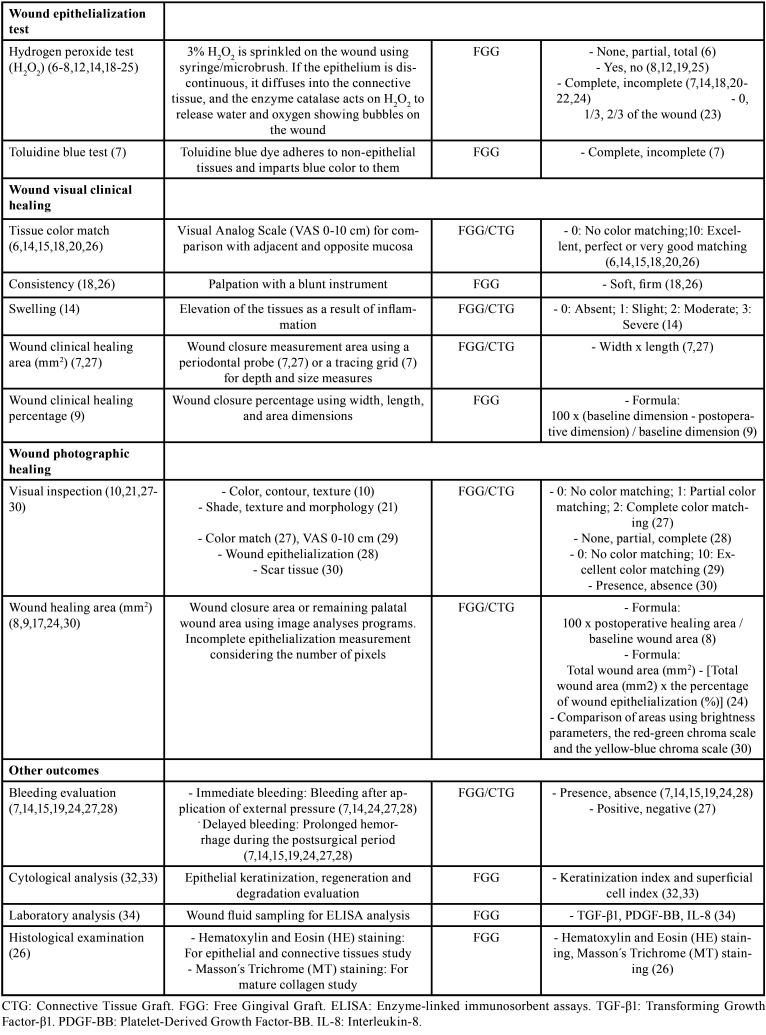
Description of outcome definitions and measures related to postoperative palatal wound healing retrieved in the literature.

Several studies provided information regarding the wound epithelialization evaluation using the hydrogen peroxide test (H2O2) (6-8,12,14,18-25) and the toluidine blue test (7) after FGG procedures. The wound visual clinical healing may be evaluated considering the tissue color match by using the Visual Analogue Scale (VAS-10 cm) for FGG and CTG procedures (6,14,15,18,20,26). In addition, consistency (18,26), and swelling (14), have been also reported as wound healing parameters for FGG or CTG. On the other hand, Pandit *et al.* (27) reported the wound closure measurement area (mm2) using a periodontal probe, whereas Sharma *et al.* (7) used a tracing grid (D-stent) for depth and size (width and length) measures after FGG or CTG procedures. Recently, Sousa *et al.* (9) analyzed the wound closure percentage after harvesting FGG using a recommended formula considering baseline and postoperative dimensions. The wound photographic healing can be evaluated through visual inspection of clinical photographies (10,21,27-30), considering characteristics of color, contour, and texture (10); shade, texture, and morphology (21); color match (27,29); wound epithelialization (28); or scar tissue (30). Likewise, several authors reported the wound healing area (mm2) or the remaining palatal wound area measured on clinical photographs using image analysis programs after FGG or CTG procedures (8,9,17,24,30). Recently, Patarapongsanti *et al*. (8) and Isler *et al.* (24) recommended calculation formulas.

Another outcome measurement evaluation reported for FGG or CTG procedures is the bleeding evaluation (7,14,15,19,24,27,28). This condition is evaluated as immediate bleeding (bleeding after application of external pressure) and delayed bleeding (prolonged hemorrhage during the postsurgical period). Finally, methods related to cytological analysis (32,33), laboratory analysis (34), and histological examination (26) have been considered as outcome measurements after FGG procedure.

Among the methods for outcome measurements evaluation related to postoperative patient-reported morbidity ( Table 2), the organized articles were distributed considering pain perception, analgesic consumption, discomfort sensation, burning sensation, changes in feeling habits, sensibility disorders, stress, quality of life, and additional questions asked to the patients using questionnaires. Pain perception is one of the most reported methods used for FGG or CTG procedures evaluation. The patient informs his/her perception using the VAS-10 cm (6,8-12,14,15,17,18,20,21,24), five-point Verbal Rating Scale (VRS-5) (7,27), 101-point Numerical Rating Scale (NRS-101) (10), and three-point Verbal Descriptor Scale (VDS-3) (28). The number of analgesic consumptions is informed considering hours, days or weeks (6,14,19-21,24,25,31,36,38). Reportedly, the discomfort sensation may also be informed using the VAS-10 cm (9,14,19,24,31,38), or the four-point Verbal Rating Scale (VRS-4) (10), whereas the burning sensation generally is informed using the VAS-10 cm (14,18,20,24,26,29). The changes or variations in feeling habits means changes in quality (liquid, soft or hard), and temperature of the food (cold, tepid or warm) (12,28), or inability to chew (31), and the VAS-10 cm is generally used (14,19,24). Kecelli *et al.* (6), Ozcan *et al.* (12), Femminella *et al.* (19), Pandit *et al.* (27), and Del Pizzo *et al.* (28) reported the sensibility disorders evaluation (sensation or sensory loss) using VDS-3 (6,12,27,28) or VAS-10 cm (19), whereas Zucchelli *et al.* (31) reported the stress associated with the level of apprehension and fear experienced for the patient of jeopardizing the palatal wound. The aforementioned outcome measurements have been referred for FGG or CTG procedures. Finally, Ozcelik *et al.* (38) reported the impact of the patient’s quality of life using the Oral Health Impact Profile-14 (OHIP-14), whereas the two investigations of Tavelli *et al.* (36,37) focused on additional questions about the use of more painkillers after the surgery and his/her wish for repeat the palatal harvesting procedure if necessary. These two outcome measurements were reported for FGG procedure.

**Table 2:**
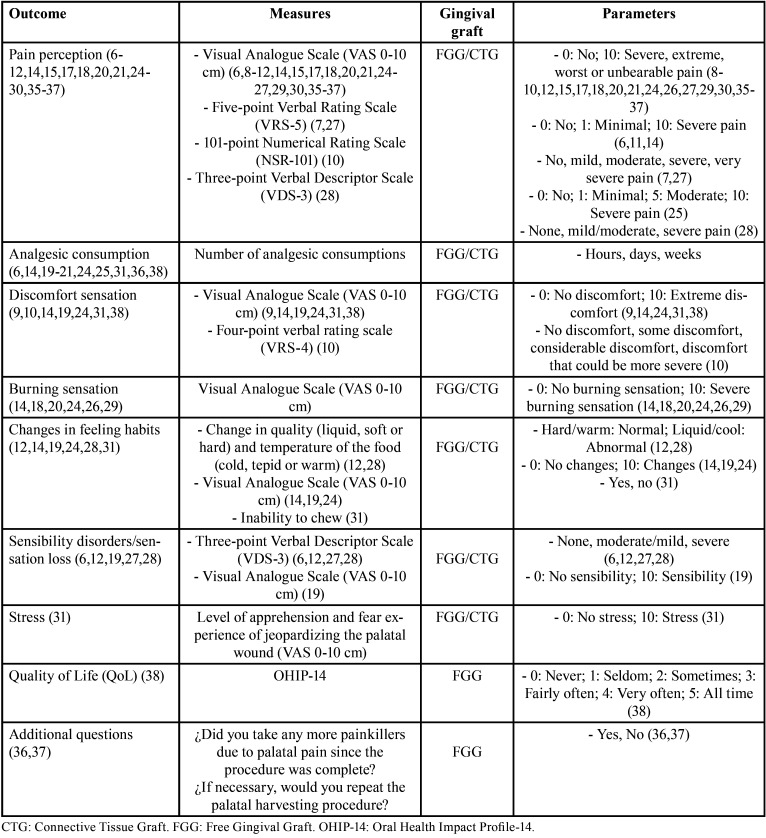
Description of outcome definitions and measures related to postoperative patient-reported morbidity retrieved in the literature.

## Discussion

Free gingival graft and CTG are two effective harvesting techniques performed on the palatal mucosa for periodontal and peri-implant plastic surgeries. Both techniques might be criticized due to patient morbidity. Therefore, it is of utmost importance that post-operative outcomes are included both in research reports as well as are assessed in clinical practice. Several methods to accelerate the healing process and to reduce postoperative pain and bleeding have been reported, but have not been organized as outcome measurements evaluated on clinical trials. The present study collects information about these outcomes in order to fill the lack of information.

In the present review, the methods for outcome measurement evaluation following FGG or CTG procedures have been addressed. The modified EHI and the modified Landry WHI are two important indexes frequently used for evaluating the palatal wound healing after FGG or CTG harvesting. The hydrogen peroxide test is commonly used as a wound epithelialization test due to practicality and does not produce color stain on the superficial tissues as it occurs when the toluidine blue test is used. In addition, clinically, professionals could make objective evaluations based on tissue color match, consistency or swelling as well as healing areas and healing percentages considering proposed formulas. Wound epithelialization evaluated by means of photographs is considered a subjective method using visual inspection or wound healing areas performed using analysis programs. It would be interesting for consecutive evaluations, minimizing memory bias. Bleeding evaluation is an important method used immediate after the procedure and in postoperative controls, specially at early follow-ups. The cytological, laboratories and histological analyses were very little reported but are important methods for healing evaluation after FGG procedure, especially in research.

Patient-centered outcome measurements are also very important and represent a true outcome in clinical practice and research. It is important to highlight that pain perception, discomfort sensation, burning sensation, changes in feeding habits, sensibility disorders, and stress evaluation require numerical or verbal rating scales such as VAS-10 cm, VDS-3, VRS-5, NRS-101 or VRS-4 to register patient experience after FGG or CTG procedures. Although several numerical and verbal scales are currently used to assess pain intensity, it remains unclear which provides the most replicable, precise, and predictive valid measurement. Jensen *et al.* (39) compared the measurement of clinical pain intensity using VAS, NRS-101, 11-point Box Scale (BS-11), 6-point Behavioral Rating Scale (BRS-6), VRS-4, and VRS-5. These authors concluded that all these scales showed predictive validity, thus were recommended for clinical use.

Several clinical trials reported different primary outcomes related to palatal wound healing after FGG procedure considering epithelialization (7,9,19,21), clinical healing (9,21), and remaining palatal wound area evaluation (24). Meanwhile, postoperative pain and discomfort sensation (7,9,19,24), alteration of sensitivity, changes in feeling habits, consumption of analgesics (19,24), and burning sensation (21,24), have been reported as secondary outcomes. On the other hand, Ehab *et al.* (25) and Tavelli *et al.* (36) considered the postoperative pain perception as a primary outcome, whereas the re-epithelialization (25,36), postsurgical bleeding, the analgesic consumption (25) and the question about willingness to repeat the treatment have been considered as secondary outcomes (36). In addition, Shanmugan *et al.* (26) considered the consistency and the color match such as objective outcomes, whereas the pain and the burning sensation were considered as subjective outcomes. Based on these findings, it may be argued that the methods related to the palatal wound healing evaluation are reported in clinical trials and could be considered as primary outcomes evaluated after FGG or CTG procedures. Considering the other group of variables described in the present review, Zucchelli *et al.* (31) included postoperative pain, discomfort sensation, bleeding, stress, and inability to chew as outcomes evaluated for patient morbidity. In this regard, Isler *et al.* (24) considered postoperative pain, discomfort sensation, changes in feeling habits, and burning sensation.

In the present review, several clinical trials evaluated the effect of platelet concentrates such as platelet-rich fibrin (8,10,12,15,19), advanced platelet-rich fibrin (9), and titanium-platelet rich fibrin (20) on the palatal wound healing and patient morbidity. Interestingly, all these clinical trials demonstrated promising results facilitating wound healing and reducing postoperative morbidity and discomfort.

Although different outcome measurements have been described in the present review, the methods used for outcome evaluation following FGG or CTG harvesting are focused considering a combination of palatal wound healing evaluation and patient morbidity evaluation. In this review, the use of the grouped outcomes is recommended for clinicians that perform periodontal and peri-implant plastic surgeries in order to adequately control the postoperative conditions of the patients. Future research about palatal healing using periodontal dressings could analyze the effect. In addition, following this type of review, additional studies could focus on methods for outcome variable evaluation after third molar extraction.

Moreover, it is clear from a research perspective, that trials should evaluate adverse events in clinical trials and also patient-centered outcomes. This is a recommendation of the CONSORT statement (40). Also, a considerable part of the evidence provided in clinical trials relates to decrease in morbidity and increase in patient satisfaction. This should be also part of clinical practice and the information coming from the present review adds possibilities of evaluation by clinicians.

## Conclusions

The most commonly used outcome measurements related to postoperative palatal wound healing are hydrogen peroxide test, tissue color match, visual inspection, and bleeding evaluation. In addition, pain perception, analgesic consumption, discomfort sensation, burning sensation, and changes in feeling habits are the most commonly used outcome measurements related to postoperative patient-reported morbidity. These outcome measurements should be part of clinical evaluation in the field.
